# Preliminary study on comparison of egg extraction methods for development of fetal bovine serum substitutes in cultured meat

**DOI:** 10.1016/j.fochx.2024.101202

**Published:** 2024-02-08

**Authors:** Da Young Lee, Dahee Han, Seung Yun Lee, Seung Hyeon Yun, Juhyun Lee, Ermie Mariano, Yeongwoo Choi, Jin Soo Kim, Jinmo Park, Sun Jin Hur

**Affiliations:** a Department of Animal Science and Technology, Chung-Ang University, Anseong 17546, Korea; b Division of Animal Science, Division of Applied Life Science (BK21 Four), Institute of Agriculture & Life Science, Gyeongsang National University, Jinju 52828, Republic of Korea

**Keywords:** FBS substitute, Serum-free media, Egg extracts, Cultured meat, Cell proliferation

## Abstract

Fetal bovine serum (FBS) substitution remains one of the challenges to the realization of cultured meat production in the marketplace. In this study, three methods were developed to extract a substitute for FBS using egg white extract (EWE): using 25 mM CaCl_2_/2.5 % ammonium sulfate/citric acid (A); ethyl alcohol (B); and 5 % ammonium sulfate/citric acid (C). B EWE can effectively replace up to 50 % of FBS in growth media (10 % of the total). Ovalbumin in the extracts can promote cell proliferation, and components along the 12 kDa protein band have the potential to inhibit cell proliferation. Chick primary muscle cells applied with B EWE, an edible material that improved the cost and time efficiency of cultured meat production, effectively proliferated/differentiated. Therefore, EWE extracted using ethyl alcohol may be used as an FBS substitute to reduce animal sacrifices and should be considered a viable alternative to FBS for cultured meat.

## Highlights

• Chick muscle primary cells can be cultured as an edible FBS substitute (EWE).
• An egg extraction method using ethyl alcohol was developed to produce a EWE.
• EWE effective for proliferation and differentiation of chicken satellite cells.

## 1 Introduction

Cultured meat is an emerging technology that has been proposed as an alternative to future meat production. Although the idea of *in vitro* meat for human consumption was conceived in the early 1930s, the most remarkable milestone was reached in 2013 when Mark Post introduced the world's first cultured meat patty (Post, 2014, Zhu et al., 2022). However, several challenges remain in realizing the successful technological transition of cultured meat to the marketplace. One particularly problematic aspect is the use of fetal bovine serum (FBS) as a component of the meat cell culture media. FBS is extracted from the blood of unborn calves found when slaughtering pregnant cows. The slaughter of pregnant livestock and its implications for animal welfare are ethically contentious issues that have received international media attention (Nielsen and Hawkes, 2019, Weber and Wagner, 2021). Additionally, FBS is a notoriously unsustainable and biologically variable ingredient, which poses safety concerns such as the potential risk of contamination by endotoxins, pathogens, and viruses. Unlike animals, a complete immune system cannot be formed during cell culture, so there is a high possibility that bacteria, mold, mycoplasma, and other pathogens will grow (Fang et al., 2017, Hanson and Ranney, 2020, Kolkmann et al., 2020). FBS is made up of thousands of substances, but its composition is not clearly known. Although FBS prices have increased approximately 300 % in the United States over the past few years, many researchers rely on FBS due to its effectiveness in cell culture systems (Fang et al., 2017, Kolkmann et al., 2020). These are some of the reasons why cultured meat does not reach the consumer market as a food product. In light of these challenges, FBS replacement technology has become an important task not only in the field of cultured meat research but also in general cell and tissue culture research (van der Valk, 2022).

Over the past few years, various serum replacements and serum-free media have been explored (Andreassen et al., 2020, Chelladurai et al., 2021, Dong et al., 2023, Kim et al., 2023, Lee et al., 2022, Lee et al., 2022, Lee et al., 2023, Okamoto et al., 2020, Sasse et al., 2000). However, existing papers mainly contain research data on serum-free media using cell lines, but research papers on serum substitutes using animal primary cells are limited (Mizunoya et al., 2015, Musa et al., 2014, Okamoto et al., 2020). Eggs contain ovalbumin (OVA), ovotransferrin (OVF), ovomucoid, lysozyme, ovoglobulin, and ovomucin, making them a useful source of nutrients for cell culture media to promote cell proliferation (Kaipparettu et al., 2008, Mizunoya et al., 2015). Bovine serum albumin, an abundant ingredient in FBS, prevents the toxicity of free fatty acids on cells in culture and improves cell growth (Keenan et al., 2006, Rodrigues et al., 2010). Thus, the egg-derived components, particularly albumin, could be effective in culturing chick muscle satellite cells. The use of egg materials to replace FBS could bring cultured meat one step closer to entering the actual food market. Additionally, the developed FBS substitute has the potential to effectively grow both cell lines and primary animal cells. The purpose of this study is to develop an efficient egg white extract manufacturing method that can proliferate and differentiate chick muscle satellite cells by replacing FBS in the industrialization of cultured meat.

## 2 Materials and methods

### 2.1 Obtaining fertilized egg white extract (EWE) according to extraction method

**Fig. 1:**
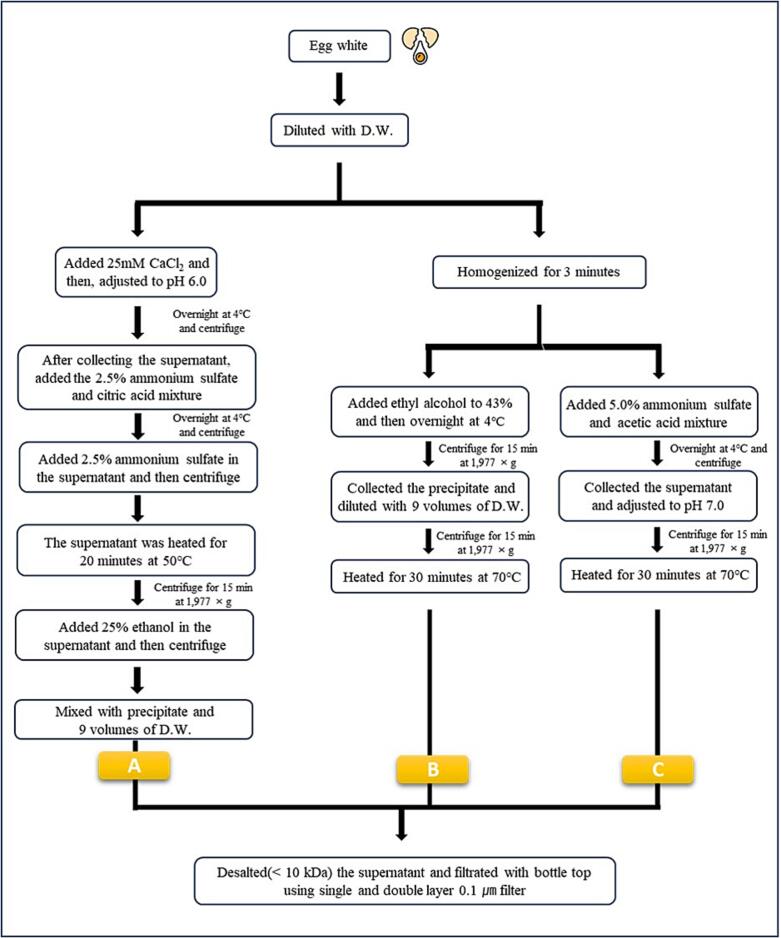
Schematic diagram of fertilized egg white extraction.

Fertilized egg white was extracted by supplementing previously specified methods (Abeyrathne et al., 2013, Ji et al., 2020). Three different methods were devised (A, B, and C) depending on the type of reagent used (Fig. 1). In a common process, egg white was separated from fertilized eggs and then mixed with distilled water at a ratio of 1:1. The full protocols are described below.

#### 2.1.1 Method A: ammonium sulfate + citric acid extraction method

25 mM CaCl_2_ was added to an amount equal to 25 % of the diluted sample, and the pH was adjusted to 6.0. The solution was reacted at 4 °C overnight and then centrifuged (1,977 × g, 15 min, 4 °C). The supernatant was collected and adjusted to pH 3.0 by adding a solution containing a mixture of 2.5 % ammonium sulfate and citric acid. After reaction at 4 °C overnight, the supernatant was collected by centrifugation (1,977 × g, 15 min, 4 °C) and reacted at 50 °C for 20 min, then diluted to contain 25 % ethanol and re-centrifuged (1,977 × g, 15 min, 4 °C). In this process, the precipitate was recovered, and residual ethanol was removed by adding distilled water, nine times the volume.

#### 2.1.2 Method B: ethyl alcohol extraction method

The sample was homogenized for 3 min using a homogenizer, and ethyl alcohol was added to 43 % (v/v). After reacting overnight at 4 °C, the sample was centrifuged (1,977 × g, 15 min, 4 °C), and the precipitate was recovered. Distilled water was added to the precipitate at nine times the volume and reacted at 70 °C for 30 min. The mixture was centrifuged again to recover the supernatant.

#### 2.1.3 Method C: ammonium sulfate + Acetic acid extraction method

The sample was homogenized for 3 min using a homogenizer. A solution containing 5 % (v/v) ammonium sulfate and acetic acid was added, and the mixture reacted at 4 °C overnight. After centrifugation (1,977 × g, 15 min, 4 °C), the supernatant was recovered, adjusted to pH 7.0, and reacted at 70 °C for 30 min. This solution was centrifuged again.

Each of the prepared extracts was purified using an Amicon centrifugal filter tube (Ultra-15 Centrifugal Filter Unit, Merck, MRK, Germany) of less than 10 kDa. The recovered supernatant was filtered through a membrane filter (0.1 μm) and stored at −20 °C until further use.

### 2.3 Chick satellite cell culture

The chick satellite cells were isolated from fertilized eggs on day 15 according to our previously published papers (Lee et al., 2021, Yun et al., 2023). The thigh part of the chick is separated and the muscle is extracted. Then, use a scalpel to mince the tissue, and centrifuge it at 1,977 × g for 3 min. Based on tissue weight after supernatant removal, enzymes digestion using collagenase D, dispase II and react at 37 °C for 30 min. The enzymatically treated tissue is sequentially filtered through a cell strainer 100, 70, and 40 µm to remove connective tissue, fat, and blood cells. Centrifuge at 1,977 × g for 3 min, and incubate in a non-coated dish in a 5 min, 37 °C, 5 % CO_2_ incubator. Only the supernatant is recovered, and the primary cells are incubated in 37 °C, 5 % CO_2_ for 3 days.

For culture experiments, the 0.1 mg/mL collagen solution was applied to cover the dish and left for 30 min. After removing the solution, the coated dish was completely dried and washed twice with 1X DPBS. Chick satellite cells were cultured in a growth medium that contained Ham's F-10 medium (LM009, Welgene, Korea) mixed with 20 % FBS (Corning, NY, USA) and 1 % penicillin/streptomycin (Gibco, MT, USA) (500 mL basal media + 100 mL FBS + 5 mL penicillin/streptomycin). The extract was prepared by completely replacing FBS (100 mL egg white extracts), replacing only 50 % (50 mL FBS + 50 mL egg white extracts), or reducing FBS by half and adding 20 % extract (50 mL FBS + 100 mL egg white extracts), an equal amount of premixed medium was dispensed into the dish. Cells were secured by seeding at a concentration of 4,000/cm^2^. Cells were incubated at 37 °C with 5 % CO_2_, and the medium was replaced every 3 days.

### 2.4 Differentiation of chick satellite cells

The chick satellite cells are replaced with a differentiation medium that consists of 2 % HS and 1 % P/S in Dulbecco’s Modified Eagle Medium (DMEM, SH30243.01, Cytiva, USA) if they reach sufficient density (>70 %). To replace the medium, remove the culture medium and wash with 1X DPBS. After washing, they will be replaced with a differentiation medium, and muscle cells will be cultured for 72 h at 37 °C in a 5 % CO_2_ incubator. Differentiation media was incubated at a dose of 150 μL/cm^2^ with rotation every 3 days.

### 2.5 Analysis of cell viability by 3-(4,5-dimethylthiazol-2-yl)-2,5-diphenyltetrazolium bromide (MTT) assay

Chick muscle satellite cells were seeded at 5 × 10^3^ cells/well in a collagen-coated 96-well plate and cultured by incubation at 37 °C in an atmosphere with 5 % CO_2_ for 24 h. Thiazolyl blue tetrazolium bromide (M2128, Sigma-Aldrich, MO, USA) was diluted with basal media (Ham's F-10) and added to a 96-well plate at a final concentration of 0.5 mg/mL (MTT solution) then incubated at 37 °C with 5 % CO_2_ for 4 h. Next, the existing solution was removed, 100 μL of dimethyl sulfoxide was added to each well, and the plate was covered and returned to the incubator for another 30 min. Subsequently, the sample absorbance was measured at 540 nm using a microplate reader (SpectraMax 190, Molecular Devices, CA, USA).

### 2.6 Determining content of EWEs using sodium dodecyl sulfate–polyacrylamide gel electrophoresis (SDS-PAGE)

SDS-PAGE was performed to investigate the protein contents of EWE with reference to Meyer and Lamberts (1965). The analysis treatment included three types of EWE according to the extraction method (A, B, C), as well as FBS, OVA, and OVF. Before SDS-PAGE, each sample was quantified by BCA Protein Assay Kits (23225, Thermo Fisher Scientific, MA, USA). The amount of protein in all treatments was adjusted to be 5 μg/well, and then the samples were reacted with 4X Laemmli sample buffer (Bio-Rad, CA, USA). Protein markers (Spectra™ Multicolor Broad Range Protein Ladder, Thermo Fisher Scientific) and samples were loaded on a 12 % acrylamide separating gel and 5 % acrylamide stacking gel by applying 80 V for 90 min. Proteins in the samples were stained with Brilliant Blue R (Sigma-Aldrich).

### 2.7 Identification of differentiated myogenesis using fluorescence dye

The immunofluorescence staining process was carried out with reference to the method of Yun et al. (2023). The primary antibody was incubated overnight with mouse monoclonal anti-MF20 antibody (1:500) on a shaker at 70 rpm at 4 °C, and the secondary antibody (1:1000) was incubated for 1 h. The nuclei were also stained with 1:1000 of Hoechst 33,342 in 1X PBS. Using the prepared dye solution, staining was performed at room temperature for 30 min. After which, all the staining solution was removed, rinsed twice, and observed under an inverted fluorescence microscope. Captured images were analyzed using ImageJ software to evaluate the degree of cell differentiation.

### 2.8 Statistical analysis

SPSS 22.0 program (IBM Corp., Armonk, NY, USA) was used to create all charts and perform the statistical analysis. Data were statistically analyzed by one-way analysis of variance (ANOVA), followed by the Tukey test for comparisons at the significance level of *p* < 0.05.

## 3 Results and discussion

### 3.1 Analysis of protein components among fertilized EWE

The three extraction methods developed in this study focused on obtaining simpler and faster extracts by complementing previous extraction methods for separating OVF and OVA from egg whites (Abeyrathne et al., 2013, Ji et al., 2020). The initial sample used was the crude extract because it was anticipated to be a good interaction between extract components that were not completely purified. The three extraction methods in Fig. 1 were designed to obtain optimal EWE by adjusting the amount of reagent, reaction time, and method based on the crude extract acquisition method.

**Fig. 2:**
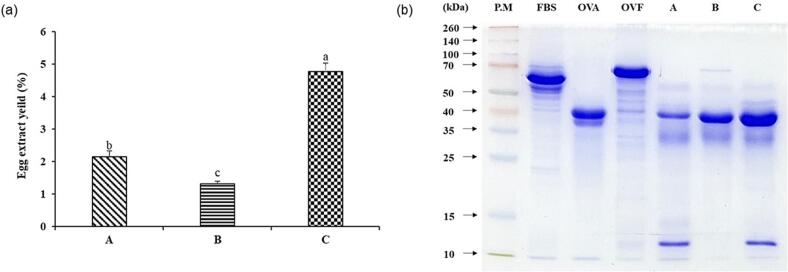
SDS-polyacrylamide gel electrophoresis (SDS-PAGE) patterns of proteins in egg white extracts (EWEs) according to the extraction method. PM: Protein marker, OVA: Ovalbumin (45 kDa), OVF: Ovotransferrin (77 kDa); A: EWE obtained using ammonium sulfate + citric acid, B: EWE obtained using ethyl alcohol, C: EWE obtained using ammonium sulfate + acetic acid. Data are given as mean ± SD (n = 3). Different letters represent significant differences between means (*p* < 0.05).

After BCA analysis of the EWEs from various extraction methods, their protein profiles were broadly determined using SDS-PAGE (Fig. 2). The result compared the extracts by adding FBS, which plays an important role in the cell growth medium, along with OVA and OVF. OVA is a major protein with a molecular weight of 42–45 kDa, accounting for approximately 54 % of egg white (Guha et al., 2019, Zhang et al., 2020). OVF is the second most abundant component (12 %) and has a molecular mass of 76–77 kDa (Abeyrathne et al., 2013, Guha et al., 2019).

Method A yielded EWE containing 1.21 mg of protein, equivalent to 2.15 % on a dry egg white basis (Fig. 2a). Method A is most similar to existing extraction methods and takes approximately 57 h (Abeyrathne et al., 2013, Ji et al., 2020). The protein profile of extract A (extracted using method A) revealed two distinct bands around 12 and 40 kDa, and a relatively cloudy and wide band was confirmed below 35 kDa (Fig. 2b). The band around 40 kDa was in a similar position to the OVA band, and the band appearing at about 12 kDa was similar in size to cystatin (12.7 kDa), one of the egg white components (Guha et al., 2019, Hunaiti et al., 2020, Mizunoya et al., 2015).

Method B yielded EWE containing 2.47 mg of protein, equivalent to 1.31 % on a dry egg white basis (Fig. 2a). The time required for method B was approximately 17 h, reducing the extraction time by 70 % and extraction cost by approximately 45 % compared to method A. In the protein profile of extract B, the band presumed to be OVA was relatively thick, so the cell proliferation effect can be expected to be prominent (Mizunoya et al., 2015).

Method C yielded EWE containing 15.93 mg of protein, equivalent to 4.78 % on a dry egg white basis (Fig. 2a). Method C also takes approximately 17 h and reduces the extraction time by 70 % and extraction cost by approximately 77 % compared to method A. In the protein profile of extract C, the band estimated to be OVA (42–45 kDa) was the thickest, and at the same time, it can be assumed to be the band most similar to G2 globulin (35–40 kDa) among egg components (Guha et al., 2019, Mizunoya et al., 2015). Additionally, a band (12 kDa) appeared at the same location as that found in extract A.

### 3.2 Culture of chick muscle satellite cells using EWE

**Fig. 3:**
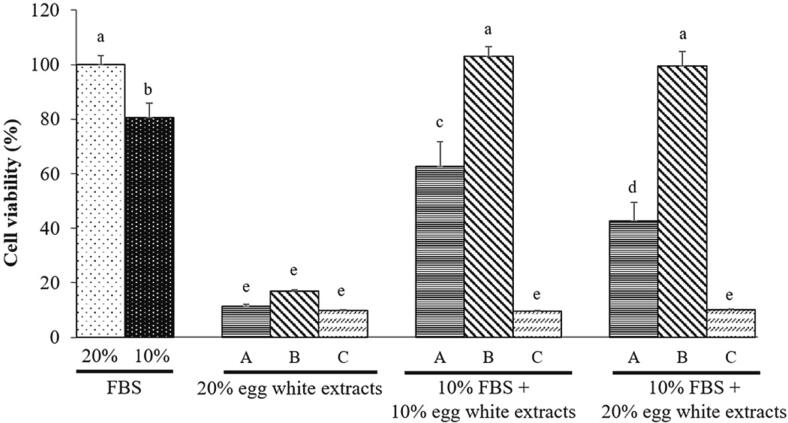
Comparison of cell viability (%) of chick muscle satellite cells in the presence of egg white extract (EWE) according to the extraction method. A: EWE obtained using ammonium sulfate + citric acid, B: EWE obtained using ethyl alcohol, C: EWE obtained using ammonium sulfate + acetic acid. Data are given as mean ± SD (n = 3). Different letters represent significant differences between means (*p* < 0.05).

**Fig. 4:**
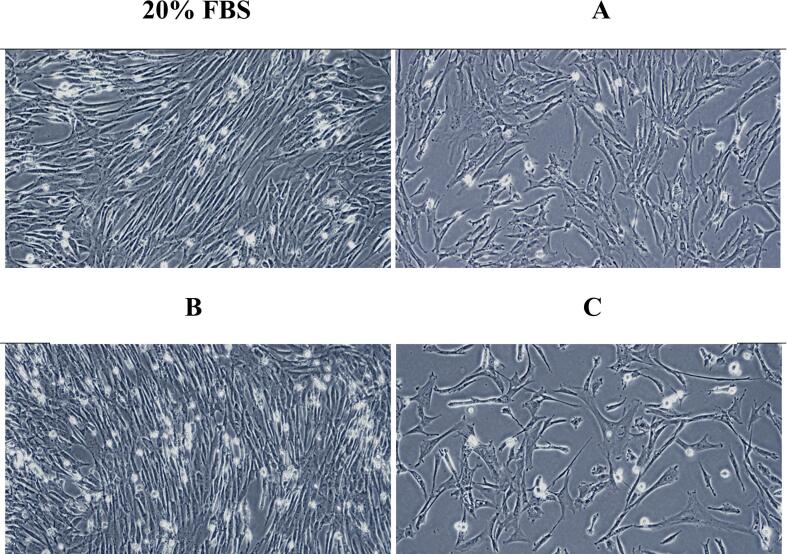
Comparison of cell morphology of chick muscle satellite cells cultured in the presence of egg white extract (EWE) according to extraction method. A: 10 % FBS + 10 % EWE obtained using ammonium sulfate + citric acid, B: 10 % FBS + 10 % EWE obtained using ethyl alcohol, C: 10 % FBS + 10 % EWE obtained using ammonium sulfate + acetic acid.

Based on the above results, we applied the extract to cell culture. Cell viability was significantly greater (*p* < 0.05) in the control group (20 % FBS treatment), treatment with only 20 % EWE was not effective, regardless of extraction of method used (Fig. 3). When Extract B was added with 10 % FBS, it had a similar effect on cell viability as 20 % FBS treatment (Fig. 3). The cell proliferation effect of extract C was not confirmed regardless of whether FBS was added (Fig. 3). Cell morphology also showed that the method B had similar cell density and shape to the control group (Fig. 4). Taken together, when EWE obtained by extraction method B is administered with 10 % FBS, it can effectively compensate for the absence of FBS and contribute to cell growth and viability.

**Fig. 5:**
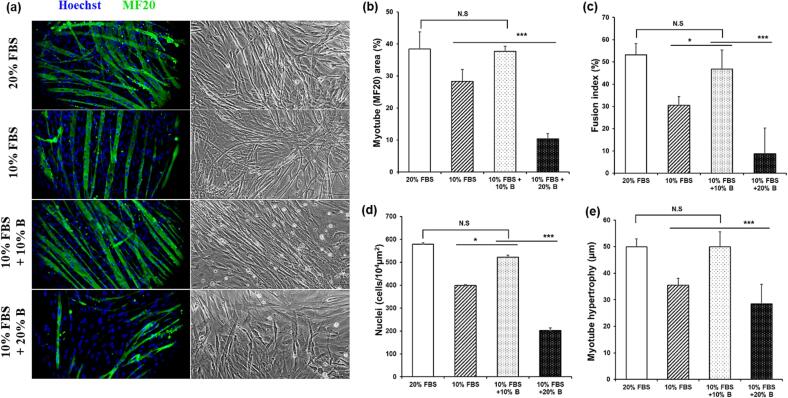
Effect of chick muscle cell differentiation treated with egg white extract using ethyl alcohol. (a) Representative image of myotubes co-stained with MF20 (MHC:green) and Hoechst:blue. (b) Indices of myotube area. (c) Indices of myotube fusion index. (d) Indices of nuclei number. (e) Indices of myotube hypertrophy. Data are expressed as the means ± SEM. n.s., not significant. * *p* < 0.05, ** *p* < 0.0001. (For interpretation of the references to colour in this figure legend, the reader is referred to the web version of this article.)

To confirm the differentiation of chick muscle cells grown with B EWE, we proceeded with differentiation step by incubating in differentiation media for 72 h. The differentiation degree of chick muscle satellite cells was measured through immunofluorescence staining 3 days after fusion. Treatment of 10 % B EWE with 10 % FBS showed an excellent replacement effect by forming chick myotubes similar to the control group (20 % FBS) (Fig. 5). This addition had a significant effect on myotube area (*p* < 0.001), fusion index (*p* < 0.05), nuclei number (*p* < 0.05), and muscle hypertrophy (*p* < 0.001) compared to the treatment group in which the FBS content was reduced by 50 % without the extract (10 % FBS) (Fig. 5). When 20 % B EWE was treated with 10 % FBS, significantly less cell differentiation was confirmed in all respects than the 10 % FBS + 10 % B EWE treatment group (*p* < 0.001), and the addition of excessive extract rather It showed a negative effect (Fig. 5).

Although the exact components of extraction method B on the EWE are not clear, the cell viability might be affected by the structural stability and nutraceutical carrier ability of egg white protein induced by ethanol or iron supplementation from OVF. Yao, Jiang, and Chen (2020) suggested that the addition of ethanol caused egg white protein aggregation, and ethanol-induced egg white gel with nutraceuticals showed well stability and carrier ability. Kleemann, Zink, Selmer, Smirnova, and Kulozik (2020) also reported that the high ethanol concentrations (≥ 60 %) increased the hardness because of the influence of solvent exchange on the ion concentration in the gels. Previous studies (Galla et al., 2021, Ko and Ahn, 2008) found that the iron-bound holo-OVF (30 μg/mL), which is stable against proteolytic hydrolysis and heat denaturation, obtained by 43 % ethanol increased the cell viability of GTL-16 and Caco-2 cells. In a study by Mizunoya et al. (2015), addition of OVA promoted the growth of C2C12 myoblasts and myotubes. OVA significantly increased the diameter of differentiated myotubes (Mizunoya et al., 2015). In a study on bovine embryo production, OVA was found to be an effective protein source for cell growth, enabling the production of bovine embryos in the absence of FCS or BSA (Tetzner et al., 2011). However, the single effect was weaker than that of FCS or BSA, and it was assessed that supplementation with growth factors, vitamins, or hormones was necessary to compensate for OVA effect (Tetzner et al., 2011). These results are similar to our results in which no cell growth effect was confirmed in the EWE treatment group alone (Fig. 3). A presumptive OVF band was identified in EWE by method B compared to other extracts, but a significantly lighter band appeared when compared to the FBS band (Fig. 3). It is very unlikely that this would have influenced the excellent effect of EWE by method B on cell proliferation. EWEs using Methods A and C, which contain a substance estimated to be cystatin, did not have a good FBS replacement effect (Fig. 3, Fig. 4). Cystatin reduced the proliferation of leukemia cells by causing cell apoptosis induced by oxidative stress, and also interfered with cell proliferation in lung tumor cells and breast tumor cells (Hunaiti et al., 2020, Ma et al., 2018, Završnik et al., 2017). These properties of cystatin can inhibit the proliferation of chick muscle satellite cells, possibly reducing the effectiveness of EWE. Therefore, for a clear and higher EWE effect, additional research is needed to identify and remove unuseful ingredients in extracts.

Cultured meat is a promising field that has the potential to be a new food that can be presented along with meat in the future. However, there are many difficulties in producing cultured meat for reasons such as price stabilization and food safety, and studies have been conducted to solve these (Jeong et al., 2021, Kolkmann et al., 2020). This study used unfertilized eggs to replace FBS, one of the culture medium compositions for culturing muscle satellite cells. Eggs are very cheap, commonly consumed food, and can be said to be a safe material as a culture medium. In particular, extraction method B is an economical approach that reduces the extraction time by approximately 70 % and extraction cost by approximately 45 % compared to the existing method (method A). Therefore, the newly developed egg material extract in this study is expected to be used as a cell culture medium and showed potential as a substitute for FBS. Furthermore, additional study should define and remove components of EWE that specifically appear in treatments with poor growth effects (extraction methods A and C). Replacing experimental ingredients such as FBS would be a way to provide consumers with safer, edible cultured meat products.

However, eggs are also a material that can only be obtained by raising animals, and their production can be affected by factors such as avian influenza or skyrocketing feed prices. Additionally, since the unfertilized egg extract material developed in this study did not show a cell proliferation effect when used alone without FBS, additional research will be needed to to completely replace FBS.

## 4 Conclusion

In this study, we developed a method to extract FBS substitutes from egg whites, and confirmed the effects on proliferation and differentiation chick muscle satellite cells. The optimal extraction method was than of using ethyl alcohol, and when added together with 10 % FBS, it showed a similar effect as 20 % FBS on the growth of chick satellite cells. The specific components of egg white, OVA and OVF, had no direct effect on cell proliferation. However, both proteins are believed to indirectly affect FBS replacement factors in chick muscle satellite cells for cultured meat development. It was speculated that a specific protein component (12 kDa) derived from egg white had a negative effect on cell proliferation. As a result, the developed egg white extraction method was able to obtain effective ingredients for the proliferation and differentiation of chick muscle satellite cells at a low price in a short period of time. Replacing cell culture materials with raw food materials is a meaningful achievement that contributes to food safety for the industrialization of cultured meat. However, further research on detailed ingredients are still neededto to completely replace FBS.

## CRediT authorship contribution statement

**Da Young Lee:** Formal analysis, Methodology, Writing – original draft, Writing – review & editing. **Dahee Han:** Investigation, Methodology, Writing – original draft, Writing – review & editing. **Seung Yun Lee:** Formal analysis, Writing – review & editing. **Seung Hyeon Yun:** Formal analysis, Writing – review & editing. **Juhyun Lee:** Resources, Visualization. **Ermie Jr. Mariano:** Resources, Visualization. **Yeongwoo Choi:** Resources, Visualization. **Jin Soo Kim:** Resources, Visualization. **Jinmo Park:** Resources, Visualization. **Sun Jin Hur:** Conceptualization, Writing – review & editing.

## Declaration of competing interest

The authors declare that they have no known competing financial interests or personal relationships that could have appeared to influence the work reported in this paper.

## Data Availability

The authors are unable or have chosen not to specify which data has been used.
